# Transcatheter arterial embolization versus surgery for refractory non-variceal upper gastrointestinal bleeding: a meta-analysis

**DOI:** 10.1186/s13017-019-0223-8

**Published:** 2019-02-01

**Authors:** Antonio Tarasconi, Gian Luca Baiocchi, Vittoria Pattonieri, Gennaro Perrone, Hariscine Keng Abongwa, Sarah Molfino, Nazario Portolani, Massimo Sartelli, Salomone Di Saverio, Arianna Heyer, Luca Ansaloni, Federico Coccolini, Fausto Catena

**Affiliations:** 1 Emergency Surgery Department, Maggiore Hospital of Parma, University of Parma, Parma, Italy; 2 Surgical Clinic, Department of Experimental and Clinical Sciences, University of Brescia, Brescia, Italy; 3 Department of Surgery, Macerata Hospital, Macerata, Italy; 4 Department of Surgery, Addenbrooke’s Hospital, Cambridge University Hospitals NHS Foundation Trust, Cambridge, UK; 5 Sidney Kimmel Medical College, Thomas Jefferson University, Philadelphia, PA USA; 6 General, Emergency and Trauma Surgery Department, Bufalini Hospital, Cesena, Italy

**Keywords:** Abdominal emergency surgery, Complicated peptic ulcer, Embolization, Meta-analysis

## Abstract

**Background:**

Nowadays, very few patients with non-variceal upper gastrointestinal bleeding fail endoscopic hemostasis (refractory NVUGIB). This subset of patients poses a clinical dilemma: should they be operated on or referred to transcatheter arterial embolization (TAE)?

**Objectives:**

To carry out a systematic review of the literature and to perform a meta-analysis of studies that directly compare TAE and surgery in patients with refractory NVUGIB.

**Materials and methods:**

We searched PubMed, Ovid MEDLINE, and Embase. A combination of the MeSH terms “gastrointestinal bleeding”; “gastrointestinal hemorrhage”; “embolization”; “embolization, therapeutic”; and “surgery” were used ((“gastrointestinal bleeding” or “gastrointestinal hemorrhage”) and (“embolization” or “embolization, therapeutic”) and “surgery”)). The search was performed in June 2018. Studies were retrieved and relevant studies were identified after reading the study title and abstract. Bibliographies of the selected studies were also examined. Statistical analysis was performed using RevMan software. Outcomes considered were all-cause mortality, rebleeding rate, complication rate, and the need for further intervention.

**Results:**

Eight hundred fifty-six abstracts were found. Only 13 studies were included for a total of 1077 patients (TAE group 427, surgery group 650). All selected papers were non-randomized studies: ten were single-center and two were double-center retrospective comparative studies, while only one was a multicenter prospective cohort study. No comparative randomized clinical trial is reported in the literature.

*Mortality*. Pooled data (1077 patients) showed a tendency toward improved mortality rates after TAE, but this trend was not statistically significant (OD = 0.77; 95% CI 0.50, 1.18; *P* = 0.05; *I*^2^ = 43% [random effects]). Significant heterogeneity was found among the studies.

*Rebleeding rate*. Pooled data (865 patients, 211 events) showed that the incidence of rebleeding was significantly higher for patients undergoing TAE (OD = 2.44; 95% CI 1.77, 3.36; *P* = 0.41; *I*^2^ = 4% [fixed effects]).

*Complication rate*. Pooling of the data (487 patients, 206 events) showed a sharp reduction of complications after TAE when compared with surgery (OD = 0.45; 95% CI 0.30, 0.47; *P* = 0.24; *I*^2^ = 26% [fixed effects]).

*Need for further intervention*. Pooled data (698 patients, 165 events) revealed a significant reduction of further intervention in the surgery group (OD = 2.13; 95% CI 1.21, 3.77; *P* = 0.02; *I*^2^ = 56% [random effects]). A great degree of heterogeneity was found among the studies.

**Conclusions:**

The present study shows that TAE is a safe and effective procedure; when compared to surgery, TAE exhibits a higher rebleeding rate, but this tendency does not affect the clinical outcome as shown by the comparison of mortality rates (slight drift toward lower mortality for patients undergoing TAE). The present study suggests that TAE could be a viable option for the first-line therapy of refractory NVUGIB and sets the foundation for the design of future randomized clinical trials.

**Limitations:**

The retrospective nature of the majority of included studies leads to selection bias. Furthermore, the decision of whether to proceed with surgery or refer to TAE was made on a case-by-case basis by each attending surgeon. Thus, external validity is low. Another limitation involves the variability in etiology of the refractory bleeding. TAE techniques and surgical procedure also differ consistently between different studies. Frame time for mortality detection differs between the studies. These limitations do not impair the power of the present study that represents the largest and most recent meta-analysis currently available.

## Introduction

### Definition, etiology, and epidemiology

Upper gastrointestinal bleeding (UGIB) is defined as bleeding originating proximal to the ligament of Treitz and is usually divided into two main categories: variceal and non-variceal. Hematemesis and melena are the commonest presentations. The most frequent cause of non-variceal bleeding is complicated peptic ulcer, but multiple etiologies must be taken into account: benign and malignant tumors, ischemia, gastritis, arterio-venous malformations (such as Dieulafoy lesions), Mallory-Weiss tears, trauma, and iatrogenic causes [1]. Another cause for UGIB is transpapillary hemorrhage, but this specific subset of cases goes beyond the aims of the present study.

A recent review on the epidemiology of complicated peptic ulcer disease [2] found that hemorrhage was by far the most common complication of peptic disease, with a reported annual incidence of hemorrhage in the general population ranging from 19.4 cases per 100,000 individuals to 57.0 cases per 100,000 individuals, with sample size-weighted average 30-day mortality of 8.6%. This high incidence of complicated peptic ulcer could be related to an increase in the use of ASA and NSAIDs and to the increasing number of elderly people. It is easy to understand how this complication remains a healthcare problem, with a great overall population health and financial impact.

### Vascular anatomy

Thorough knowledge of the anatomy of the supramesocolic area is of utmost importance to understand the pros and cons of angioembolization, and the likelihood of successful embolization reflects prior knowledge of the location of the bleed. A complex network of anastomotic arteries provides a rich blood supply to the upper part of the gastrointestinal tract. This can make successful embolization more challenging; however, it decreases the incidence of post-embolization ischemia [3].

Anatomic variations in the celiac anatomy, most notably in the origins of the hepatic arteries, occur in at least 50% of the population. Such variations must always be considered when evaluating a patient angiographically for NVUGIB [4].

### Historical background

Historically, the first-line therapy for NVUGIB was surgery and a myriad of different surgical approaches are described in the literature, some of which are still used today. However, surgery for bleeding peptic ulcers is associated with an 8 to 33% risk of post-operative mortality [5]. Angiography with percutaneous catheterization as a diagnostic tool for GI bleeding was first performed by Baum and colleagues in 1965, identifying hemorrhage as contrast extravasation in four out of eight patients [6]. After a couple of years, Rosch et al. [7] were the first to successfully control an acute gastric hemorrhage by embolizing the gastroepiploic artery with an autologous blood clot. Since then, embolization therapy has gained increasing popularity due to its limited physiological trauma, especially in high-risk patients unfit for surgery. Furthermore, improvements in catheter technology, the development of new materials for embolization therapy, and wider availability of appropriately skilled interventional radiologists have led to the increased use of angiography and embolization in the management of UGIB. Nowadays, angiography permits the visualization of the entire mesenteric system, thus allowing the identification of more rare sites of hemorrhage, such as the biliary tree. Its specificity approaches 100% [4], while the sensitivity remains around 60% [8, 9]. However, the implementation of a CT-angiogram, that has accuracy for active bleeding up to 89% [10], can help overcome this lack of sensitivity. Nevertheless, angiography has a specific set of complications that includes access site thrombosis or hemorrhage, contrast reactions, injury to target vessels (including dissection and distal embolization), and ischemic damage after embolization.

The introduction of proton pomp inhibitors, as well as the creation and improvement of effective endoscopic techniques, greatly changed the approach to non-variceal UGIB, shifting the therapeutic path from a surgery-first to a conservative-first approach. Nowadays, patients with NVUGIB that fail endoscopic hemostasis (refractory NVUGIB) usually have very big ulcers that are located in difficult areas and bleed from major vessels. These issues, in combination with the increase in the number of fragile, elderly patients, many of which have co-morbidities, pose a real clinical dilemma: should their NVUGIB be managed surgically, or be referred to an interventional radiologist for transcatheter arterial embolization (TAE)?

## Objectives and rationale of the research

No clear evidence regarding the role of angioembolization for the treatment of refractory NVUGIB is currently available in the literature. The aim of this study is to carry out a systematic review of the literature and to perform a meta-analysis of the studies that directly compare TAE and surgery in patients with NVUGIB. The results of the study will provide information that can assist clinicians to make the correct choice between angioembolization and surgery when managing patients with NVUGIB.

## Materials and methods

### Search methods

We searched PubMed, Ovid MEDLINE, and Embase. To achieve maximum sensitivity, a combination of the MeSH terms “gastrointestinal bleeding”; “gastrointestinal hemorrhage”; “embolization”; “embolization, therapeutic”; and “surgery” were used ((“gastrointestinal bleeding” or “gastrointestinal hemorrhage”) and (“embolization” or “embolization, therapeutic”) and “surgery”). The search was performed in June 2018. Studies were retrieved and relevant studies were identified after reading the study title and abstract. Furthermore, the bibliographies of the selected studies were examined to identify any additional relevant studies.

### Selection criteria

We used the PRISMA (Preferred Reporting Items for Systematic Reviews and Meta-Analyses) guidelines [11] to formulate the basis of eligibility criteria using the PICO (P - Populations/People/Patient/Problem, I - Intervention(s), C - Comparison, O - Outcome) worksheet and search strategy (Table 1).

**Table 1:** PICO (Patients, Intervention, Comparison, Outcome) worksheet

Population	Adult patients with refractory NVUGIB (defined as failure of endoscopic hemostasis or rebleeding after successful endoscopic hemostasis)
Intervention	Transcatheter angioembolization (TAE)
Comparison	Direct comparison of TAE and surgery; report of at list one of the considered outcomes. If multiple trials or studies were published by the same center, only the most complete one was included. Studies regarding transpapillary bleeding were excluded
Outcome	All-cause mortality with no time limit; rebleeding or continued bleeding; complications, both procedure-related and not procedure-related; need for further intervention for any reason

RCTs, case-control, and cohort studies meeting the following criteria were included: (1) English language, (2) considering adult patients’ population with refractory NVUGIB (defined as failure of endoscopic hemostasis or rebleeding after successful endoscopic hemostasis), (3) direct comparison of TAE and surgery, and (4) report of at least one of the considered outcomes (mortality, rebleeding, complications, need for further intervention). If multiple trials or studies were published by the same center, only the most complete one was included. Studies regarding transpapillary bleeding were excluded. Case reports, editorials, letters, and studies containing duplicate data or data already published were excluded.

### Data collection, assessment of study quality, and risk of bias

Studies were selected by two authors (A.T., Fa.C), and disagreements were resolved by collegial discussion. The full text of the included studies was obtained and reviewed, to determine the relevance and the quality of the paper. Risk of bias assessment was performed according to the Newcastle–Ottawa Quality Assessment Scale criteria.

### Statistical analysis

Statistical analysis was performed using RevMan software (Review Manager version 5.3, Nordic Cochrane Centre, Copenhagen, Denmark). Odds ratio with a 95% confidence interval was used to compare outcomes. A fixed effect model was used in case of low heterogeneity, while a random effects model was used when significant heterogeneity was noted. Heterogeneity was assessed using the Cochrane *Q* square test (*P* < 0.1 was considered an indicator of significant heterogeneity) and the *I*^2^ estimates (< 25% moderate, 25–50% moderate, > 50% high heterogeneity).

### Outcomes

The outcomes considered for analysis in the present study were all-cause mortality with no time limit, rebleeding rate, complication rate, both procedure-related and not procedure-related, and need for further intervention. Rebleeding was defined as bleeding from the same site after a successful index procedure or as a continuing bleeding, not controlled by index procedure. The need for further intervention comprehends all procedures performed after the index operation, either for a rebleeding or for a complication deriving from the index operation.

## Results

**Fig. 1:**
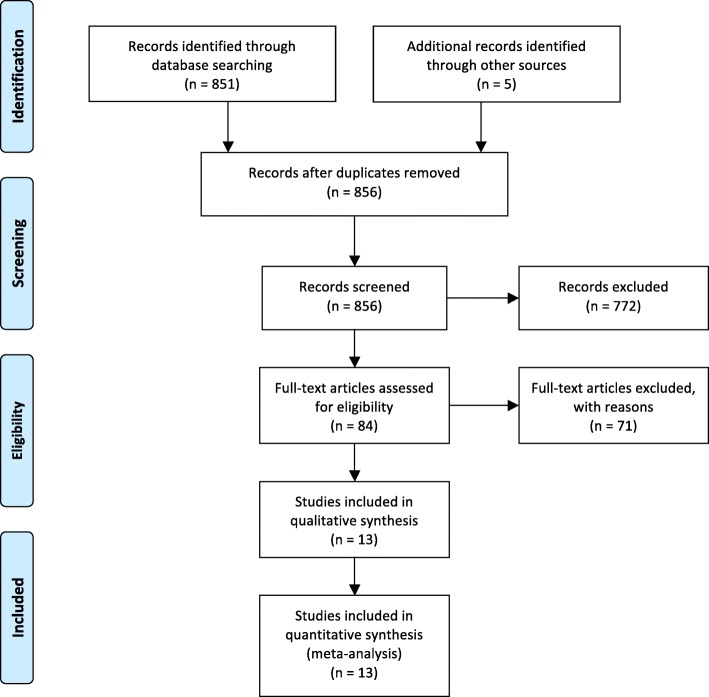
Literature search—flowchart of literature search and study selection according to the PRISMA statement

A total of 856 abstracts were found; the majority of them were excluded because they were not relevant. Of the remaining 84 studies, 71 were excluded for the following reasons: 64 did not compare TAE with surgery, one was based on a pediatric population, four did not have an English full-text, one did not provide the raw data for the statistical analysis [12], and another one did not provide data for the surgical sub-population [13]. Only 13 studies were selected [14–26] (Fig. 1, Table 2) for inclusion: all the selected papers were non-randomized studies, published between January 2004 and January 2017. Of the selected studies, 10 were single-center retrospective comparative studies, two were a double-center retrospective comparative study, and only one was a multicenter prospective cohort study. Currently, no comparative randomized clinical trial is reported in the literature.

**Table 2:** Summary of included studies

First author, year of publication	Country	Time frame	Etiology of NVUGIB	No. of patients			Study design
				TAE	Surgery	Total	
Ripoll 2004	Spain	1986–2001	Gastric–duodenal ulcers	31	39	70	Single center, retrospective
Eriksson 2008	Sweden (Uppsala)	1998–2005	Various etiologies	40	51	91	Single center, retrospective
Langner 2008	Germany	2001–2006	Various etiologies	11	17	28	Single center, retrospective
Larssen 2008	Norway	2000–2005	Various etiologies	46	51	97	Single center, retrospective
Defreyene 2008	Belgium	1993–2003	Various etiologies	36	10	46	Single center, retrospective
Venclauskas 2010	Lithuania, Sweden	2000–2007	Duodenal ulcers	24	50	74	Double center, retrospective
Wong 2011	Hong Kong	2000–2009	Gastric–duodenal ulcers	32	56	88	Single center, retrospective
Ang 2012	Singapore	2004–2010	Various etiologies	30	63	93	Single center, retrospective
Jairath 2012	UK	2007	Various etiologies	60	97	157	Multicenter, prospective
Jailani 2014	Malaysia	2006–2012	Various etiologies	24	21	45	Single center, retrospective
Laursen 2015	Denmark	1997–2013	Gastric–duodenal ulcers	45	73	118	Single center, retrospective
Griffiths 2016	Australia	2004–2012	Various etiologies	24	79	103	Double center, retrospective
Nykänen 2017	Finland	2000–2015	Gastric–duodenal ulcers	24	43	67	Single center, retrospective

### Risk of bias

The risk of bias was assessed using the Newcastle–Ottawa Quality Assessment Scale: all the included papers are of poor quality with a high risk of bias (Table 3).

**Table 3:** Newcastle–Ottawa Scale (NOS) for assessing the quality of non-randomized studies in meta-analyses — Good quality: 3 or 4 stars (*) in selection domain AND 1 or 2 stars in comparability domain AND 2 or 3 stars in outcome domain; Fair quality: 2 stars in selection domain AND 1 or 2 stars in comparability domain AND 2 or 3 stars in outcome/exposure domain; Poor quality: 0 or 1 star in selection domain OR 0 stars in comparability domain OR 0 or 1 stars in outcome/exposure domain

Study	Selection				Comparability of cohorts	Outcome			Quality/total score
	Representativeness of exposed cohort	Selection of non-exposed cohort	Ascertainment of exposure	Outcome not present at baseline		assessment of outcome	Sufficient follow-up duration	Adequate follow-up	
Ripoll	*	*	*	–	–	*	*	–	Poor/5
Eriksson	*	*	*	–	–	*	*	–	Poor/5
Langner	*	*	*	–	*	*	–	–	Poor/5
Larssen	*	*	*	–	–	*	–	–	Poor/4
Defreyene	*	*	*	–	–	*	*	–	Poor/5
Venclauskas	*	*	*	–	–	*	–	–	Poor/4
Wong	*	*	*	–	–	*	–	–	Poor/4
Ang	*	*	*	–	–	*	*	–	Poor/5
Jairath	*	*	*	–	–	*	*	–	Poor/5
Jailani	*	*	*	–	–	*	–	–	Poor/4
Laursen	*	*	*	–	–	*	–	–	Poor/4
Griffiths	*	*	*	–	–	*	*	*	Poor/6
Nykänen	*	*	*	–	–	*	–	–	Poor/4

### Population

**Fig. 2:**
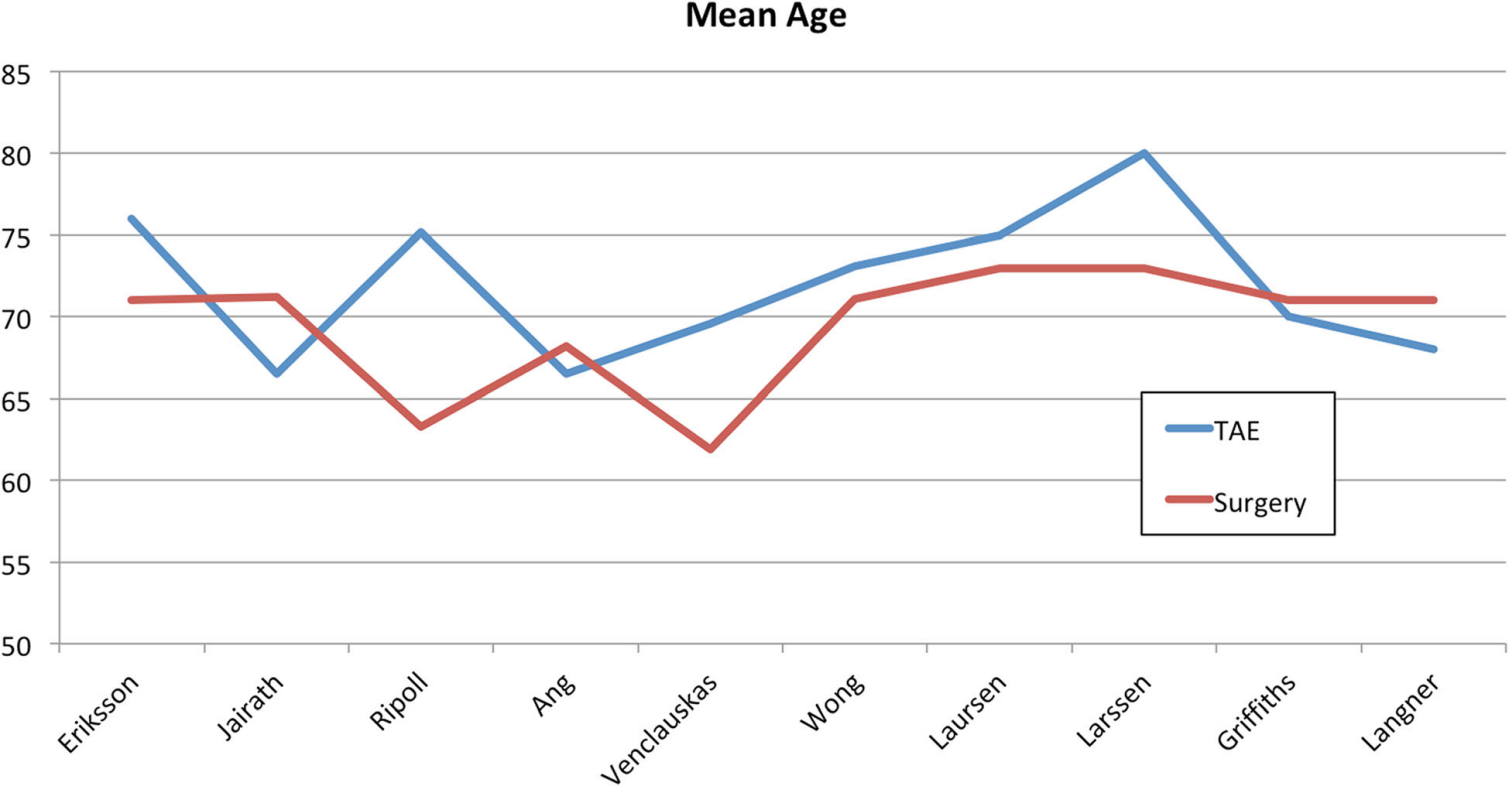
Average age — Graphical comparison of the average age of the TAE and surgery groups across included studies

A total of 1077 patients were included in the study, with 427 patients in the TAE group and 650 patients in the surgery group. Only ten of the included studies reported the mean age of the patients, and the sample size-weighted mean age is 72.2 years for the TAE group and 69.6 years for the surgery group (Fig. 2).

### Criteria for patient allocation

Only a few studies reported the indication that drove the choice between TAE and surgery. Nykänen et al. [26] decided on the base of the availability of the interventional radiologist and on the hemodynamic status, with no other specifications. According to Griffiths [25], indications for emergency surgery were uncontrollable bleeding at initial endoscopy, rebleeding following endoscopic hemostasis, proximal small bowel bleeding, and an unstable patient diagnosed with a bleeding duodenal ulcer on computed tomography angiogram; no indications were reported for TAE. Jailani and colleagues [23] referred to surgical treatment for patients with acute, life-threatening, and ongoing bleeding. Patients were referred to TAE by Eriksson et al. [15] mainly when skilled interventional radiology coverage was available and the patient was a poor surgical candidate. Langner [16] based his decision on the patients’ surgical risk factors and the overall clinical situation. Defreyene and colleagues [17] did not provide any clear criteria to refer patients to surgical or angiographic treatment, but they interestingly found that decision-making after endoscopic failure was significantly affected by the presence of a peptic ulcer. On the other hand, Ripoll and her group [14] decided on an individual basis whether the patient would benefit from emergency laparotomy or embolization therapy and embolization therapy was considered when the patient was at high surgical risk. The remaining papers did not provide any information on patients’ allocation [18–22].

### Etiology of bleeding

Only six studies focused their attention on bleeding deriving exclusively from a duodenal or gastric peptic ulcer [14, 18–20, 24, 26]. The remaining seven studies include different etiologies that cause NVUGIB (anastomotic ulcer, Dieulafoy ulcers of the stomach and duodenum, post-operative pseudo-aneurysm, angiodysplastic lesions, leiomyomas, post-sphincterectomy bleeding, duodenal and jejunal diverticuli, etc.).

### TAE techniques

Significant differences are present in the techniques and the materials utilized for angioembolization among the considered studies. Two studies did not report any data about TEA procedures [22, 24]. In the other studies, the types of embolization agents included coils, gel-foam particles, and polyvinyl alcohol particles. An evident extravasation of contrast into the bowel lumen was considered as active bleeding. In the absence of a clear contrast extravasation, prophylactic embolization was performed on the base of the endoscopic evidence or on the guide of clips placed during the first endoscopy.

### Type of surgery

According to the variability of the etiologies underlying the bleeding, surgical procedures considered in the selected studies differ remarkably. Under-running suture of the ulcer is by far the most commonly performed surgical procedure, followed by partial and total gastrectomy and truncal vagotomy with pyloroplasty. A variety of other procedures (ligation of gastroduodenal artery, small bowel resection, excision of polyp, ulcer excision, etc.) are also present and constitute a small proportion of the included cases. None of the included studies specify whether surgery was performed laparoscopically or with an open approach.

### Clinical outcome — Mortality (Fig. 3)

All thirteen studies reported the mortality rate. The definition for mortality included death from all causes and the time frame varies between studies: 30-day mortality was used in seven studies [15, 18, 20, 21, 23, 24, 26], in-hospital mortality was used in four studies [14, 17, 22, 25], and two studies did not report any time frame for mortality [16, 19]. Pooled data from all the 13 studies (1077 patients) showed a tendency toward improved mortality rates after TAE, but this trend is not statistically significant (OD = 0.77; 95% CI 0.50, 1.18; *P* = 0.05; *I*^2^ = 43% [random effects]). Furthermore, statistically significant heterogeneity was found among the studies.

**Fig. 3:**
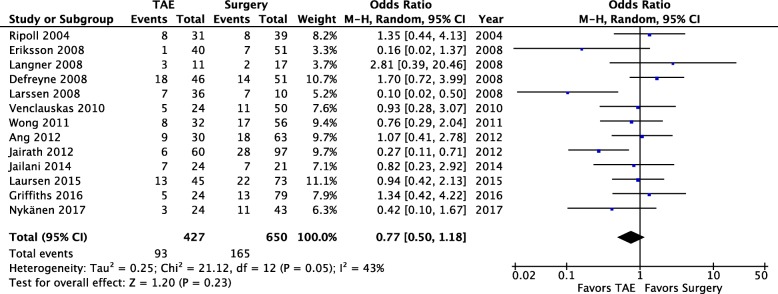
In-hospital all-cause mortality — Comparison of mortality rates rates between the two study groups, forest plot of comparison (random effects)

### Clinical outcome — Rebleeding rate (Fig. 4a, b)

Eleven out of the 13 selected studies reported a rebleeding rate. Rebleeding was defined as recurrent bleeding or as continuous bleeding after the primary procedure has been performed. The time frame for rebleeding is specified in only three studies (within 1 week from the index procedure [21]; distinction between rebleeding after 3 days and after 30 days [17]*,* and within 30 days [26]). Pooled data (865 patients, 211 events) showed that the incidence of rebleeding was significantly higher for patients undergoing TAE, compared to those who underwent surgery (OD = 2.44; 95% CI 1.77, 3.36; *P* = 0.41; *I*^2^ = 4% [fixed effects]).

**Fig. 4:**
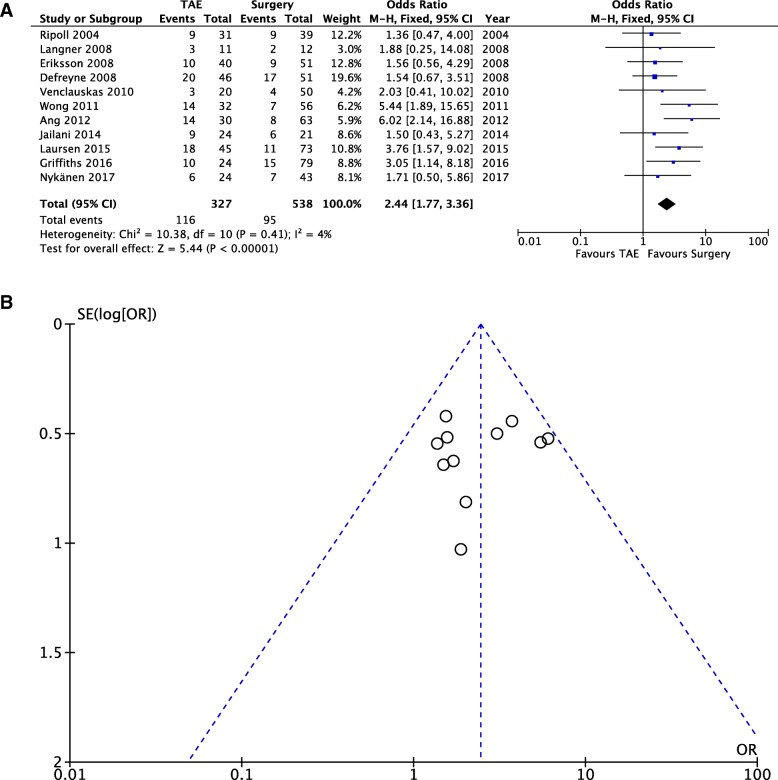
Rebleeding rates — Comparison of rebleeding rates between the two study groups (fixed effects). **a** Forest plot of comparison. **b** Funnel plot of comparison

### Clinical outcome — Complication rate (Fig. 5a, b)

The complication rate was reported in only six studies and is defined as the number of patients with at least one complication. The following subsets were included in the complication rate: TAE-related complications, surgery-related complications, and medical complications. Only major complications were included for the present meta-analysis. Only three studies [15, 20, 26] analyzed selectively TAE-related (i.e., pancreatitis, acute kidney injury, duodenal ischemia, and coil misplacement) and surgery-related complications (i.e., post-operative abscess, duodenal stump of anastomotic leakage, paralytic ileus, dehiscence of the fascia). Pooling of the data (487 patients, 206 events) showed a sharp reduction of complications after TAE compared to surgery (OD = 0.45; 95% CI 0.30, 0.47; *P* = 0.24; *I*^2^ = 26% [fixed effects]). Furthermore, no significant heterogeneity was found among the studies.

**Fig. 5:**
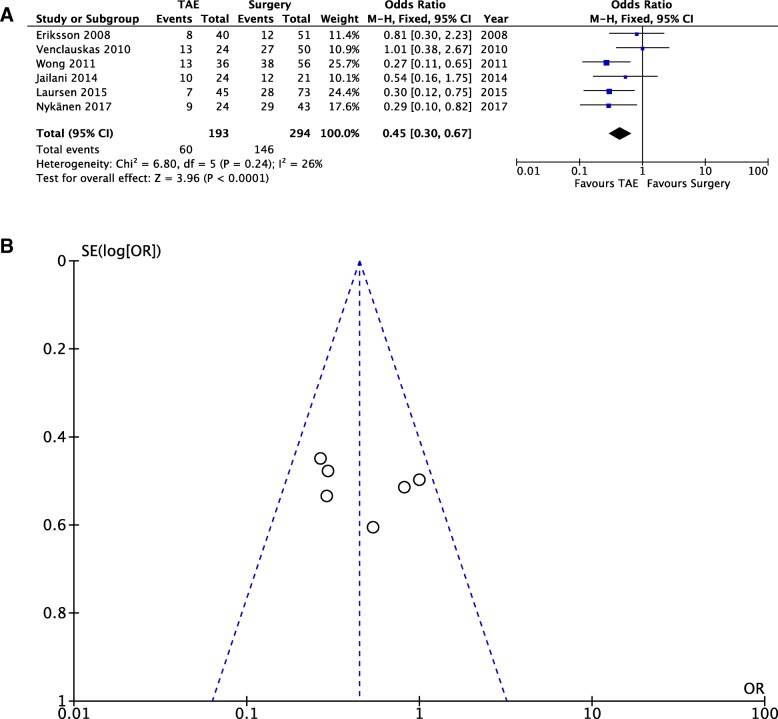
Complication rates — Comparison of complication rates between the two study groups (fixed effects). **a** Forest plot of comparison. **b** Funnel plot of comparison

### Clinical outcome — Need for further intervention (Fig. 6)

Nine studies analyzed the need for further intervention after the index procedure. This category includes every invasive procedure (mainly endoscopy, angioembolization, or surgery) needed to secure hemostasis or to treat a complication. Pooled data (698 patients, 165 events) revealed a significant reduction of further intervention in the surgery group (OD = 2.13; 95% CI 1.21, 3.77; *P* = 0.02; *I*^2^ = 56% [random effects]). A great degree of heterogeneity was found among the studies, and this could be related to a selection bias, because some of the studies did not report the need for additional intervention in the case of a procedure-related complication, but only in the case of rebleeding.

**Fig. 6:**
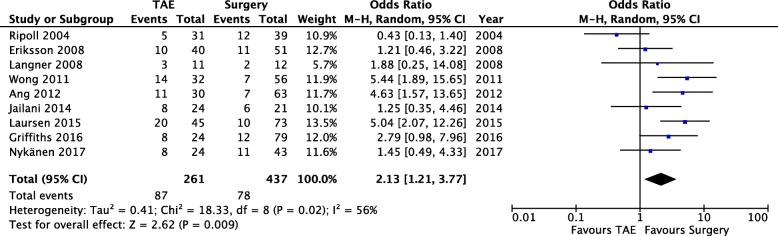
Need for further intervention — Comparison of reintervention rates between the two study groups. Forest plot of comparison (random effects)

## Discussion

In the case of non-variceal upper-GI bleeding, when medical and endoscopic treatment fails, surgery or transcatheter embolization is the available treatment option. Over the past few decades, the number of patients requiring surgical intervention has decreased enormously. In the 1990s, up to 13% of patients required surgery to control bleeding from peptic ulcer disease [27], but with improved endoscopic hemostatic techniques and intravenous proton pump inhibitor infusions, the rate of surgical procedures has dropped to less than 2% in the present day [28, 29]. In fact, endoscopic treatment is extremely effective in controlling NVUGIB, but despite adequate initial endoscopic therapy, refractory NVUGIB can occur in up to 24% of high-risk patients [30] and mortality after a surgical salvage in the recent UK National Audit was still as high as 29% [22]. The technological advances in interventional radiology are improving rapidly, whilst the experience of surgeons in the management of upper GI hemorrhage is declining. This trend is likely to continue in the future, so it is necessary to precisely determine the criteria that drive the choice between surgical and radiological treatment for NVUGIB.

In 1999, a prospective randomized study from Lau et al. [31] compared endoscopic retreatment with surgery for rebleeding after initial endoscopy and found that in patients with peptic ulcers and recurrent bleeding, endoscopic retreatment reduces the need for surgery without increasing the risk of death and is associated with fewer complications than surgery. Furthermore, identification of the source of recurrent bleeding at a second endoscopy can lead to the placement of endoscopic clips at the ulcer rim; should endoscopic treatment fail to control the hemorrhage, the metal clips could provide the interventional radiologist with useful information to identify and embolize the culprit vessel in the absence of angiographic stigmata of bleeding [32]. This implies that in the case of a bleeding peptic ulcer, surgical hemostasis or angiographic embolization should be performed after failure of repeated endoscopy. The same study from Lau [31] found that hypotension at randomization (*P* = 0.01) and an ulcer size of at least 2 cm (*P* = 0.03) were independent factors predictive of the failure of endoscopic retreatment, suggesting that patients with hypotension and/or ulcer larger than 2 cm may undergo surgery/angiography without repeated endoscopy. This finding was confirmed by Wong et al. in 2011 in a retrospective review of a cohort of 3271 patients with peptic ulcer bleeding [20].

Despite the limitations of the present study, this is the most updated and comprehensive meta-analysis, to our knowledge, that compares surgery to angioembolization for refractory NVUGIB and the results that arise from it are extremely interesting.

We found no difference in mortality rates between the two procedures, with a slight drift to a lower mortality in the TAE group (odds ratio [OD] = 0.77; 95% confidence interval [CI] 0.50, 1.18; *P* = 0.05; *I*^2^ = 43 [random effects]) (Fig. 4), despite the higher incidence of rebleeding after TAE (OD = 2.44; 95% CI 1.77, 3.36; *P* = 0.41; *I*^2^ = 4% [fixed effects]) (Fig. 5a, b). A statistically significant heterogeneity was found among the studies in mortality rates, and this could be due to the different time-frames for mortality assessment used between the different studies (30-day mortality was used in seven studies [15, 18, 20, 21, 23, 24, 26], in-hospital mortality was used in four studies [14, 17, 22, 25], and two studies did not report any time frame for mortality [16, 19]). These results are even more important given the baseline differences in the two groups: frailer, older patients with more comorbidities tended to be treated with TAE. This undeniable selection bias is related to the methods for allocation of patients in the two arms of the meta-analysis: none of the included studies provides a clear list of the indication for TAE and the decision was mostly made by the attending surgeon on an individual basis; on the basis of personal experience, availability of an interventional radiologist, and operating room; and on the basis of patient clinical conditions and comorbidities. Defreyene and colleagues [17] found that decision-making after endoscopic failure was significantly affected by the presence of a peptic ulcer: regardless of the bleeding severity or their clinical condition, patients with a peptic ulcer at endoscopy were 5.2 times more likely to be referred to surgery, and neither indirect parameters of hemodynamic instability nor coagulopathy independently influenced the choice of rescue. This bias was also reported by Beggs et al. [33] that found a statistically significant higher incidence of coagulopathy and ischemic heart disease in patients undergoing TAE. On the other hand, a recent systematic review on embolization for NVUGIB found a mean technical success rate of 84% and a mean clinical success rate of 67%; while mean rebleeding rate, mean complication rate, and mean 30-day all-cause mortality were 27, 6, and 8%, respectively [34]. The same study analyzed the factors related to angioembolization failure and found that the presence of coagulopathy and/or multi-organ failure has the worst impact on the outcome of embolization. In light of the above statement, the differences in patients’ comorbidities and coagulopathy at time of intervention between the two cohorts analyzed in the present study could then explain the higher rate of rebleeding in the TAE group. Furthermore, the study from Defreyne et al [17] showed that rebleeding after TAE was observed only in the early post-procedural period (within 3 days), while after that time period, rebleeding episodes were observed only in the surgery group. This result further strengthens the hypothesis that rebleeding rate in the TAE group could be related to temporary physiological derangements. Another aspect that can explain this higher rate of rebleeding is the intermittent nature of this kind of hemorrhage, that makes it harder to identify the bleeding site during the angiographic study and could lead to notable variations in vessel diameters, thus making the embolic agent too small to completely occlude the vessel.

The complication rate is unarguably in favor of TAE (OD = 0.45; 95% CI 0.30, 0.47; *P* = 0.24; *I*^2^ = 26% [fixed effects]) (Fig. 6a, b), even if only six of the analyzed studies reported complications. Although the upper GI tract usually has a rich collateral blood supply, previous studies have shown ischemic complications to occur in 7 to 16% of cases [35, 36] and they can either present acutely, with GI necrosis, or later, with ischemic duodenal stenosis. It is important to remember that multiple factors, such as previous surgery, pancreatitis, and radiation therapy, can interfere with collateral circulation and cause ischemia. Poultsides et al. [35] reported four (7%) cases of bowel ischemia following embolization, all of whom had surgically altered anatomy. Other known causes of ischemia include the use of embolic agents, such as liquid agents (e.g., cyanoacrylate glue) or very small particles (e.g., gelatin sponge powder) that occlude more distally in the vascular bed. Only three of the included studies [15, 20, 26] selectively analyzed TAE-related (i.e., pancreatitis, contrast-induced nephropathy, duodenal ischemia, coil misplacement, embolization of non-target vessels, access site arterial trauma, intimal dissection, or pseudo-aneurysm formation) and surgery-related complications (i.e., post-operative abscess, duodenal stump of anastomotic leakage, paralytic ileus, dehiscence of the fascia). The most accurate report is from Nykänen et al. [26] that described, out of 53 embolizations, one (1.8%) iatrogenic dissection of superior mesenteric artery, four (7.5%) acute kidney injuries with three of them requiring dialysis, four (7.5%) gastroduodenal ischemic findings at follow-up endoscopy, and one (1.8%) migrating coil protruding through the duodenal ulcer into the duodenal lumen. These numbers are probably the closest to the real incidence of TAE-related complications; the tendency toward complications under-report in the other studies could be related to lack of data or difficulties in data collection, given the retrospective nature of the studies and the wide time spread of the analyzed cases (from 1986 to 2015). The increased complication rate after surgical treatment could also explain the absence of differences in mortality rate despite the increased risk of rebleeding after TAE: the choice between TAE and surgery seems to inevitably pose the dilemma of having an increased risk of rebleeding or an increased risk of complications. Consideration of these two possibilities must be weighted for individual patients and could be the element that drives treatment strategy, given the absence of strong, evidence-based recommendations.

In 2001, Defreyne et al. found that clinical parameters such as surgery after endovascular embolization failure negatively impacted survival [37]. This result is extremely interesting and necessitates further investigation of this specific subset of patients, because it is plausible that the relative ischemia induced by a previous embolization could negatively affect the results of surgery. If this were the case, a meticulous evaluation of the rebleeding risk could be necessary and surgery as a first option could be taken into account for patients with a high rebleeding risk.

A recent retrospective study from the Karolinska Institute [12], not included in this study because it did not provide raw data for the meta-analysis, included 282 patients with a 3-year follow-up, found a significant reduction in mortality and hospital length of stay after TAE, that could be explained by the comparability in terms of age and comorbidities of patients in the surgery and TAE groups. This is a further stimulus to implement TAE into everyday management of refractory NVUGIB.

The discussion of the results for the last analyzed outcome, i.e., the need for further intervention after the index operation, is limited by the nature of the included studies. In fact, only nine of them included this outcome in their analysis but a great degree of heterogeneity was found and this could be related to a selection bias, because some of the studies did not report the need for additional intervention in the case of procedure-related complications, but only in the case of rebleeding. Nevertheless, our meta-analysis revealed a significant reduction of further intervention in the surgery group (OD = 2.13; 95% CI 1.21, 3.77; *P* = 0.02; *I*^2^ = 56% [random effects]) (Fig. 6), but this result is likely to be related to the increased rate of rebleeding found in the TAE group.

Some confusion still remains on the applications of TAE in hemodynamically unstable patients, and none of the included studies analyzed this specific subset of patients. From the existing data, it seems that physician expertise and availability of equipment are the driving factors in the decision to perform TAE on hemodynamically unstable patients. More specifically, it is likely possible to perform TAE in these patients in facilities with a 24-h availability of an expert interventional radiologist and a hybrid OR, while it is not recommended in smaller facilities with limited resources.

It is also necessary to take into account the previous surgical history of the patient, because it could significantly alter the vascular anatomy, and the presence of post-surgical adhesions could make access to the gastroduodenal area arduous and time-consuming.

### Limitations

The study has some limitations. First of all, the retrospective nature of the majority of included studies leads to an inevitable selection bias. Furthermore, the decision between TAE and surgery was made on an individual case-by-case basis by the attending surgeon, making group allocation and randomization difficult to achieve. This may lead to low external validity. Furthermore, although the most common cause of refractory bleeding is a peptic ulcer, there remain a variety of etiologies. TAE and surgical techniques also vary among the included studies. Regarding complications, only a few studies reported a complication rate for the analyzed procedures and some studies reported reintervention rates only in case of rebleeding after the index procedure; thus, the analysis of the reintervention and complication rates may not represent everyday reality. Lastly, the modality of mortality detection differs between the studies (in-hospital mortality, 30-day mortality, overall mortality, etc.). These limitations do not impair the power of the present study that represents the largest and most recent meta-analysis available.

## Conclusions

The management of NVUGIB refractory to endoscopic treatment remains a clinical dilemma and the decision between TAE and surgery is usually made on an individual basis by the attending surgeon. The aim of this meta-analysis is to gather the currently available evidence, thus providing a guide for every clinician facing this emergency. Unfortunately, the quality of the studies currently published is low and their retrospective nature increases the likelihood of selection bias.

Nevertheless, the results of the present study show that TAE is a safe and an effective procedure and, when compared to surgery, TAE has a higher rebleeding rate, but this tendency does not affect the clinical outcome. In fact, the comparison of mortality rates for the two procedures highlights a slight drift toward a lower mortality for patients undergoing TAE, despite the fact that the TAE patient population usually includes those with greater comorbidities unfit for surgery.

The present study suggests that TAE could be a viable option as a first-line therapy for refractory NVUGIB and, in the absence of evidence of superiority of one specific approach, local factors, such as organization of surgical and radiological services, availability of specific radiological skills, services availability during night shift and weekends, etc., will continue to determine the therapeutic pathway. This study also aims to set the basis for the design of future randomized clinical trials. Another issue to be addressed in the future is the best treatment option for refractory NVUGIB in hemodynamically unstable patients.

## Abbreviations

ASA: Acetylsalicylic acid
CI: Confidence interval
CT: Computed tomography
NSAIDs: Non-steroidal anti-inflammatory drugs
NVUGIB: Non-variceal upper gastrointestinal bleeding
OD: Odds ratio
OR: Operating room
PICO: Populations/People/Patient/Problem, Intervention(s), Comparison, Outcome
PRISMA: Preferred Reporting Items for Systematic Reviews and Meta-Analyses
RCTs: Randomized controlled trials
TAE: Transcatheter arterial embolization
UGIB: Upper gastrointestinal bleeding
